# Scaffold-free development of multicellular tumor spheroids with spatial characterization of structure and metabolic radial profiles

**DOI:** 10.1007/s44164-024-00074-3

**Published:** 2024-07-16

**Authors:** Shelby N. Bess, Gaven K. Smart, Matthew J. Igoe, Timothy J. Muldoon

**Affiliations:** Department of Biomedical Engineering, University of Arkansas, Fayetteville, AR USA

**Keywords:** Multicellular spheroids, Macrophage, Metabolism, Autofluorescence, Fluorescence microscopy

## Abstract

**Purpose:**

*In vitro* assays are essential for studying cellular biology, but traditional monolayer cultures fail to replicate the complex three-dimensional (3D) interactions of cells in living organisms. 3D culture systems offer a more accurate reflection of the cellular microenvironment. However, 3D cultures require robust and unique methods of characterization.

**Methods:**

The goal of this study was to create a 3D spheroid model using cancer cells and macrophages, and to demonstrate a custom image analysis program to assess structural and metabolic changes across spheroid microregions.

**Results:**

Structural characterization shows that cells at the necrotic core show high normalized fluorescence intensities of CD206 (M2 macrophages), cellular apoptosis (cleaved caspase-3, CC3), and hypoxia (HIF-1α and HIF-2α) compared to the proliferative edge, which shows high normalized fluorescence intensities of CD80 (M1 macrophages) and cellular proliferation (Ki67). Metabolic characterization was performed using multiphoton microscopy and fluorescence lifetime imaging (FLIM). Results show that the mean NADH lifetime at the necrotic core (1.011 ± 0.086 ns) was lower than that at the proliferative edge (1.105 ± 0.077 ns). The opposite trend is shown in the A1/A2 ratio (necrotic core: 4.864 ± 0.753; proliferative edge: 4.250 ± 0.432).

**Conclusion:**

Overall, the results of this study show that 3D multicellular spheroid models can provide a reliable solution for studying tumor biology, allowing for the evaluation of discrete changes across all spheroid microregions.

## Introduction

In vitro-based assays are an important component of cellular and tumor biology that have been able to provide a fast, simple, and cost-effective tool to complement large-scale animal testing. The use of 2D monolayers is one of the most commonly employed pre-clinical in vitro methodologies for drug development and studying cellular signaling (among other applications) because of their cost-effectiveness, reproducibility, and ease of handling [1–5]. However, there are limitations to monolayer cultures. Cells in the in vivo environment are surrounded by a variety of cell types (i.e., immune cells and fibroblasts) and extracellular matrix (ECM) in a three-dimensional (3D) structure; 2D culture does not fully account for the natural 3D environment that is seen in vivo [1, 6–8]. Increasing evidence has shown that 3D culture systems can accurately represent cellular microenvironments in contrast to traditional 2D monolayer cultures. More specifically, cells in 3D culture systems differ physiologically and morphologically (e.g., cellular survival, proliferation, and gene expression heterogeneity) from cells in 2D monolayer cultures. Key physiological differences include the distribution of nutrients and substrates, proper cell–cell and cell-ECM interactions that create environmental “niches”, and preserved morphology and cellular division [1, 4, 5, 9–13]. The additional dimensionality of 3D cultures is an important feature that causes differences in cellular responses because it influences the spatial organization of cells and their surface receptors that are engaged with surrounding cells. These spatial aspects within 3D culture systems affect the signal transduction of cells, ultimately influencing cellular behavior through gene expression, making these cellular responses more similar to in vivo behavior [1, 14–16]. There has been increased effort to develop various 3D culture systems and their applications in cancer biology, drug discovery, stem cell research, and other in vitro-based assays [17–23].

Along with the development of spheroid cell culture models, well-established procedures and methods must be adapted as cutting-edge technologies to adequately define these complex cellular aggregates [24, 25]. Various techniques, such as flow cytometry and Western blotting, have been used to study 3D tumor spheroid characteristics, such as (i) morphology [26, 27], (ii) topography [27, 28], (iii) size [29, 30], (iv) cellular organization [31], (v) protein and gene expression [32, 33], (vi) cell cycle patterns [34–36], and (vii) invasive and metastatic potential of cancer cells [37–41]. However, these techniques require cells to be disaggregated and suspended to study individual cell populations and proteins [34, 42]. In this situation, optical imaging techniques such as brightfield and fluorescence microscopy are useful for evaluating the size, shape, and internal structures of spheroids [4]. Furthermore, these imaging techniques allow for the observation and analysis of the spheroid internal arrangement as well as the status of the cells in each microregion. Antibodies that selectively target proteins (caspase-3, HIF, and Ki-67) or biomarkers (EF5 and pimonidazole) are used to assess the cellular microenvironment (hypoxia) or conditions (proliferation, senescence, or apoptosis). Furthermore, fluorescence microscopy may be used to perform fluorescence-based live/dead assays on 3D spheroids to detect the distribution of dead and living cells. Though these methods have been employed in the analysis of multicellular spheroids, most of these techniques are often tailored for monolayer cultures, meaning that universal experimental procedures for analyzing 3D cell cultures have yet to be devised [43]. To increase the number of tools/techniques used to analyze 3D culture methods, the goals of this study aim to create a i) 3D in vitro culture system that can reproducibly create multicellular tumor spheroids; ii) develop suspension-based immunocytochemistry techniques and high-resolution imaging protocols used to quantify cellular populations and spheroid structures along with monitor changes in NADH and FAD autofluorescence; and iii) create a custom image analysis program to characterize holistic structural and metabolic changes across spheroid micro-regions using radial line intensity profiles.

## Methods

### Cancer cell and macrophage culture

Murine RAW 264.7 (ATCC^©^, TIB-71) and CT26 colorectal adenocarcinoma (ATCC^©^, CRL-2638) cancer cells were maintained in Roswell Park Memorial Institute (RPMI)-1640 medium (Invitrogen™, 10,104-CV) supplemented with 10% fetal bovine serum (FBS) (ATCC^©^, 30–2020TM) and 1% gentamicin (Invitrogen™, 15710064). All cells were grown to ~ 80% confluence with passage number remaining under ten prior to the 3D multicellular spheroid culture.

### 3D multicellular spheroid culture

**Fig. 1:**
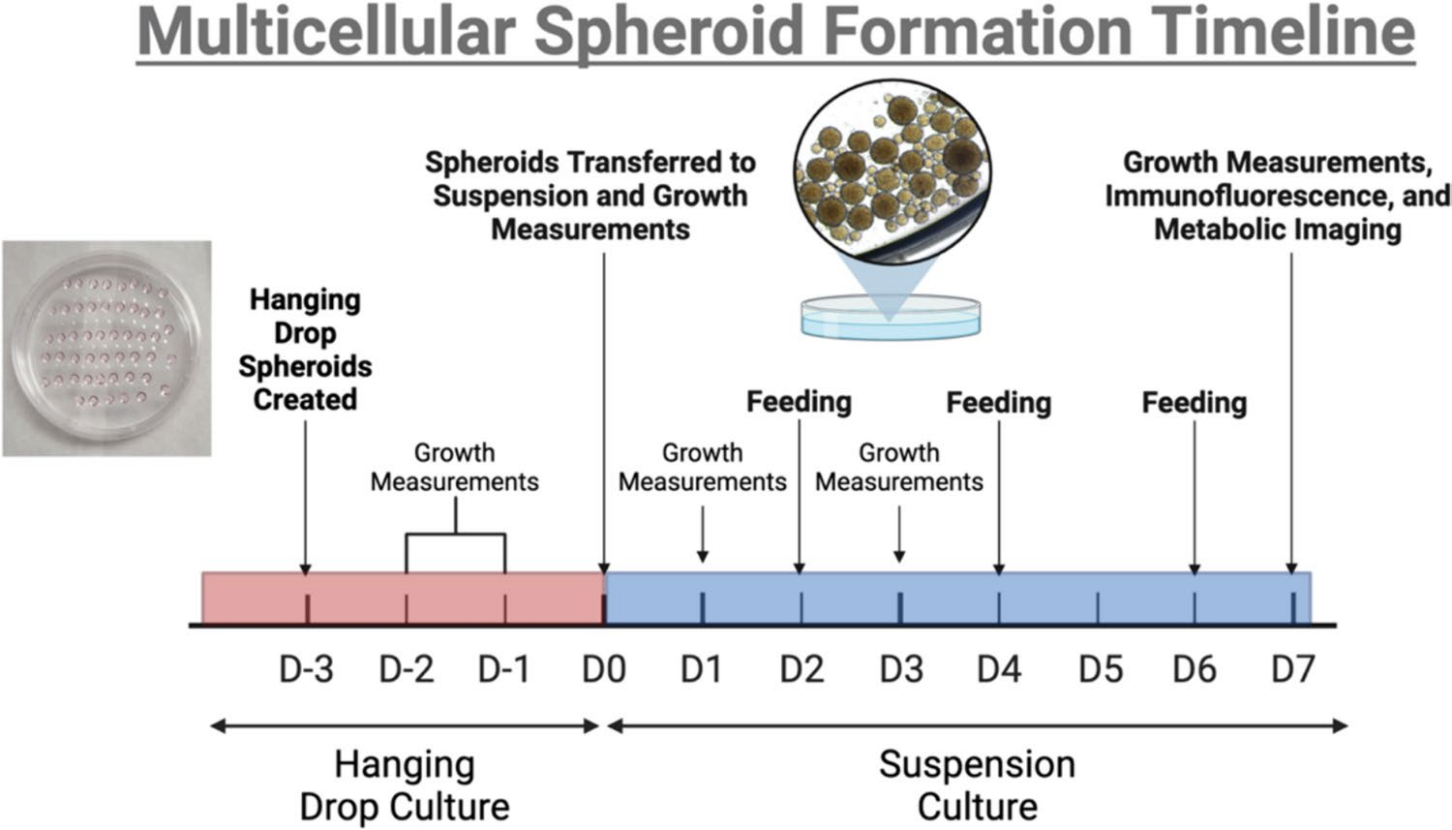
Schematic of 3D multicellular spheroid culture. Briefly, murine CT26 colorectal adenocarcinoma cells and RAW 264.7 macrophages were brought to a concentration of 2 × 10^6^cells/mL and 1 × 10^6^cells/mL, respectively. A mixed cell suspension was created and 20 µL hanging drops were deposited onto an inverted petri dish lid. The lid was then inverted onto a petri dish filled with 10 mL of PBS. The petri dish was placed on an orbital shaker at 70 RPM for 3 days. After 3 days, the multicellular spheroids were washed from the petri dish lid and centrifuged prior to being transferred to a 35 mm dish. Spheroids were placed on the orbital shaker and allowed to culture for an additional seven days with feeding occurring every two days. Figure was created in BioRender

RAW 264.7 macrophages were brought to a concentration of 1 × 10^6^ cells/mL, and CT26 cancer cells were brought to a concentration of 2 × 10^6^ cells/mL. A combined cell suspension of macrophages and cancer cells was created before spheroid formation. On an inverted 100 mL petri dish lid, 20 μL hanging drops (*n* = 50 ± 5) of the cell suspension were deposited on the inner side of the lid. The lid was inverted and placed on top of the bottom of a petri dish filled with 10 mL of PBS [44]. Dishes were placed on an orbital shaker at 70 RPM (ThermoFisher™, 88881101) for 3 days in a 37 °C incubator at 5% CO2 (Days -3 to -1) [45, 46]. The hanging drops were then washed with RPMI culture medium and centrifuged at 1000 RCF for 1 min. The supernatant was removed, and new media were added prior to transfer to a 35 mm MatTek dish (Day 0) (Fig. 1). The spheroids were placed on an orbital shaker at 70 RPM in a 37 °C incubator at 5% CO_2_ for an additional 7 days to allow spheroid formation. Spheroids were fed every two days, by collecting using a wide-orifice micropipette tip, and centrifuged at 1000 RCF for 1 min. Supernatant was removed, and new media were added prior to spheroid transfer to a new 35 mm MatTek dish before being placed on the orbital shaker at 70 RPM in a 37 °C incubator at 5% CO_2_.

### Growth curve measurements

Growth curves were created to assess spheroid growth during two culture methods: (i) hanging drop and (ii) suspension. Spheroids were imaged with a wide-field upright microscope (Nikon, Eclipse Ci), 4X/0.13NA objective lens (Nikon, CFI Plan Fluor 10X), digital camera (Nikon, DS-Fi2), and PC-based camera control unit (Nikon, DS-U3). For the hanging drop culture, each hanging drop was imaged each day (Days -3 through Day 0). For suspension culture, a total of ten field-of-view (FOV) were captured on Days 1, 3, and 7. The diameter of each spheroid within each FOV was measured using ImageJ software. Experiments were performed in triplicate, with n = 100 spheroids at each time point.

### Immunofluorescence staining and imaging

Immunofluorescence staining was performed to characterize individual cell populations and the structural micro-regions within the multicellular spheroid model. To quantify macrophage populations, Brilliant Violet 421™ CD80 primary antibody (Biolegend®, 104725) was used to detect CD80, a M1 macrophage marker, while an AlexaFluor 594 anti-mouse CD206 primary antibody (Biolegend®, 141726) was used to detect CD206, a M2 macrophage marker. To quantify the cellular proliferation, a primary antibody was used to detect Ki67 (ThermoFisher®, PA5-19462) with an AlexaFluor 488 secondary antibody (ThermoFisher®, A11034) A primary antibody that targets cleaved caspase 3 (CC3) (Cell Signaling Technology®, 9664S) along with an AlexaFluor 594 secondary antibody (ThermoFisher®, A11012) was used to quantify cellular apoptosis. It is known that within the necrotic core, hypoxia can develop due to the lack of nutrients and the diffusion of oxygen. A FITC conjugated primary antibody (Fisher Scientific®, PIMA545251) was used to quantify HIF-1α while a DyLight™ 650 conjugated primary antibody (Fisher Scientific®, PIPA522694) was used to quantify HIF-2α. All staining was performed in suspension within a microcentrifuge tube [47]. Briefly, spheroids were collected using a wide orifice micropipette tip and centrifuged at 1000 RCF for 1 min. Spheroids were then washed with PBS and centrifuged at 1000 RCF for 1 min. 10% neutral buffered formalin was added to the spheroids and allowed to incubate at room temperature for 30 min. Spheroids were then washed three times with PBS and centrifuged at 1000 RCF for 1 min between each wash. 0.2% Triton-X100 was added to the spheroids and allowed to incubate at room temperature for 15 min. Spheroids were washed three times with PBS and centrifuged at 1000 RCF for 1 min between each wash. Prior to antibody addition, 2% bovine serum albumin (BSA) was added to the spheroids and allowed to incubate for 60 min at room temperature. Primary antibodies (conjugated and non-conjugated) were added and allowed to incubate overnight at 4 °C. After the overnight incubation, for CD80/CD206 and HIF-1α/HIF-2α staining, spheroids were washed with PBS and added to a glass slide and mounted with Fluoromount G and a coverslip. For Ki67/CC3 staining, secondary antibodies were added to the spheroids and allowed to incubate for 60 min. Spheroids were then washed with PBS and added to a glass slide and mounted with Fluoromount G and a coverslip. Images were acquired with a wide-field upright microscope (Nikon, Eclipse Ci), 10X/0.3NA objective lens (Nikon, CFI Plan Fluor 10X), digital camera (Nikon, DS-Fi2), and PC-based camera control unit (Nikon, DS-U3). Experiments were performed in triplicate with *n* = 85 ± 15 spheroids imaged.

### Live-spheroid metabolic imaging

Prior to imaging, a separate group of unstained spheroids were moved to a microincubator with a controllable temperature and humidified gas delivery (5% CO_2_). A custom inverted multiphoton imaging system (Bruker custom system) equipped with an Ultrafast Ti:Sapphire (Mai Tai HP, Spectra Physics, Inc.) via a (25x/1.1NA) water immersion objective with a 2 mm working distance (Olympus) and four close-proximity high-efficiency GaAsP detectors. NADH fluorescence was captured with a 460 (± 20) nm bandpass filter at 755 nm excitation, and FAD fluorescence was captured with a 525 (± 25) nm bandpass filter at 855 nm excitation. NADH and FAD fluorescence were normalized by PMT gain and laser power, with PMT gain normalized to fluorescein concentrations, as in previous studies [48, 49]. PMT gain and laser power were maintained constant, and the laser power was read after each imaging session. An integrated fluorescence lifetime imaging microscopy (FLIM) module was used to measure mitochondrial function with respect to the different components contributing to NADH autofluorescence. Before the autofluorescence and FLIM images were acquired, the z-axis was adjusted across all channels to ensure that a cross section was captured. Experiments were performed in triplicate with *n* = 85 ± 15 spheroids imaged.

### Pre-processing of immunofluorescence and multiphoton images

**Fig. 2:**
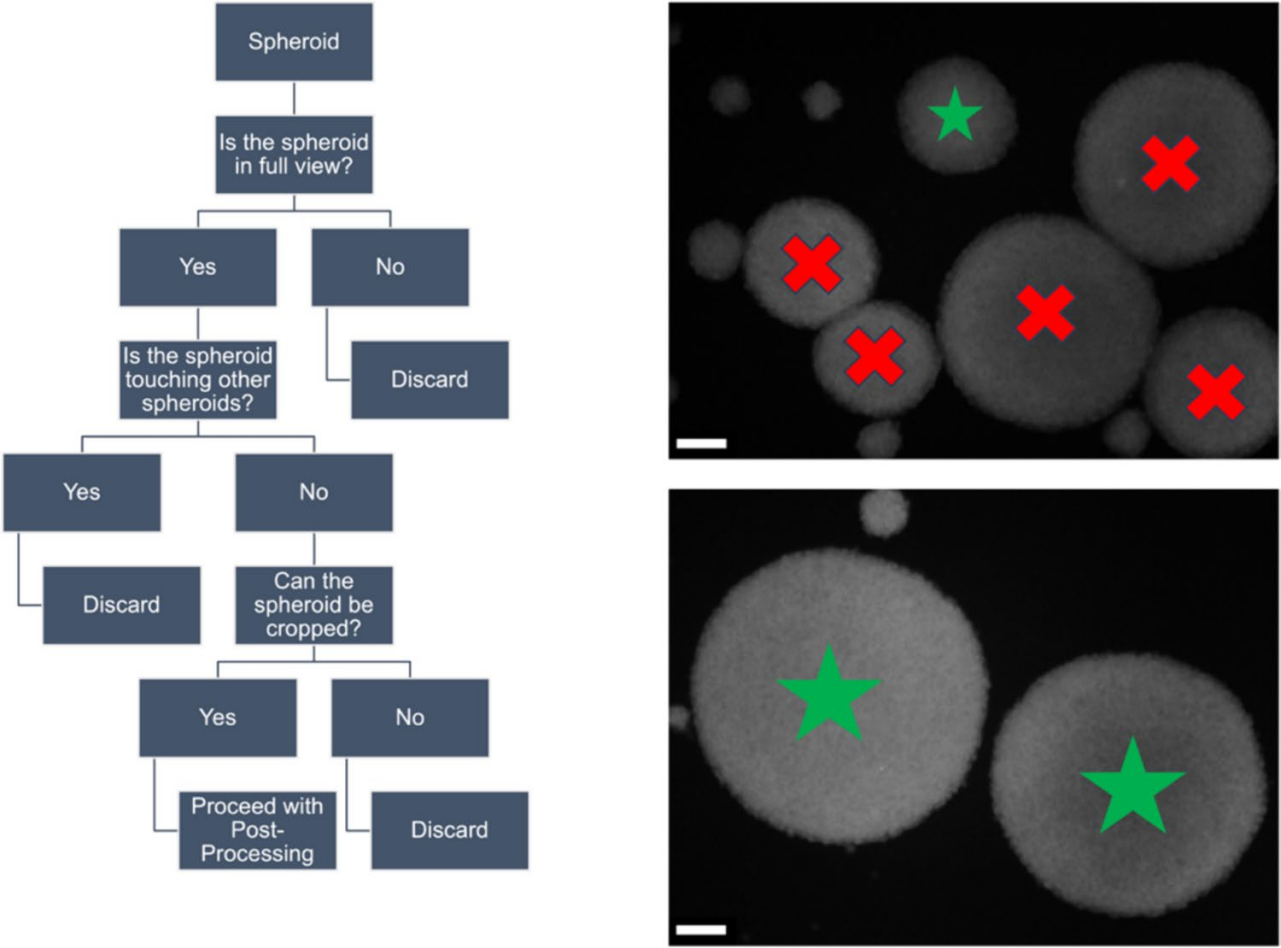
Flowchart describing the pre-processing scheme. Right panels: Representative images of spheroids that were acceptable (Green star) or unacceptable (Red X) for post-processing using the radial line profile script. Scale bars are 50 µm

Pre-processing must be performed before processing all immunofluorescence and multiphoton images. Figure 2 shows a flow chart describing the preprocessing of the spheroids. Briefly, once an image was opened, all spheroids within it were counted and logged. Individual spheroids were also analyzed. First, it is necessary to determine whether the spheroid is in full view within the image. If the spheroid was not fully visible, it was discarded. If the spheroid is in full view, it must be determined whether the spheroid is touching other spheroids within the field of view. If the spheroid touched other spheroids, it was discarded. If the spheroid does not touch other spheroids, it must be determined whether the spheroid can be cropped for further processing in the radial line profile script. If the spheroid could not be cropped, it was discarded. If a spheroid can be cropped, one can proceed with i) image processing using a radial line profile script (immunofluorescence and NADH/FAD intensity images) or ii) bi-exponential modeling to measure the decay curve of NADH fluorescence intensity (FLIM images).

### Radial line profile program

**Fig. 3:**
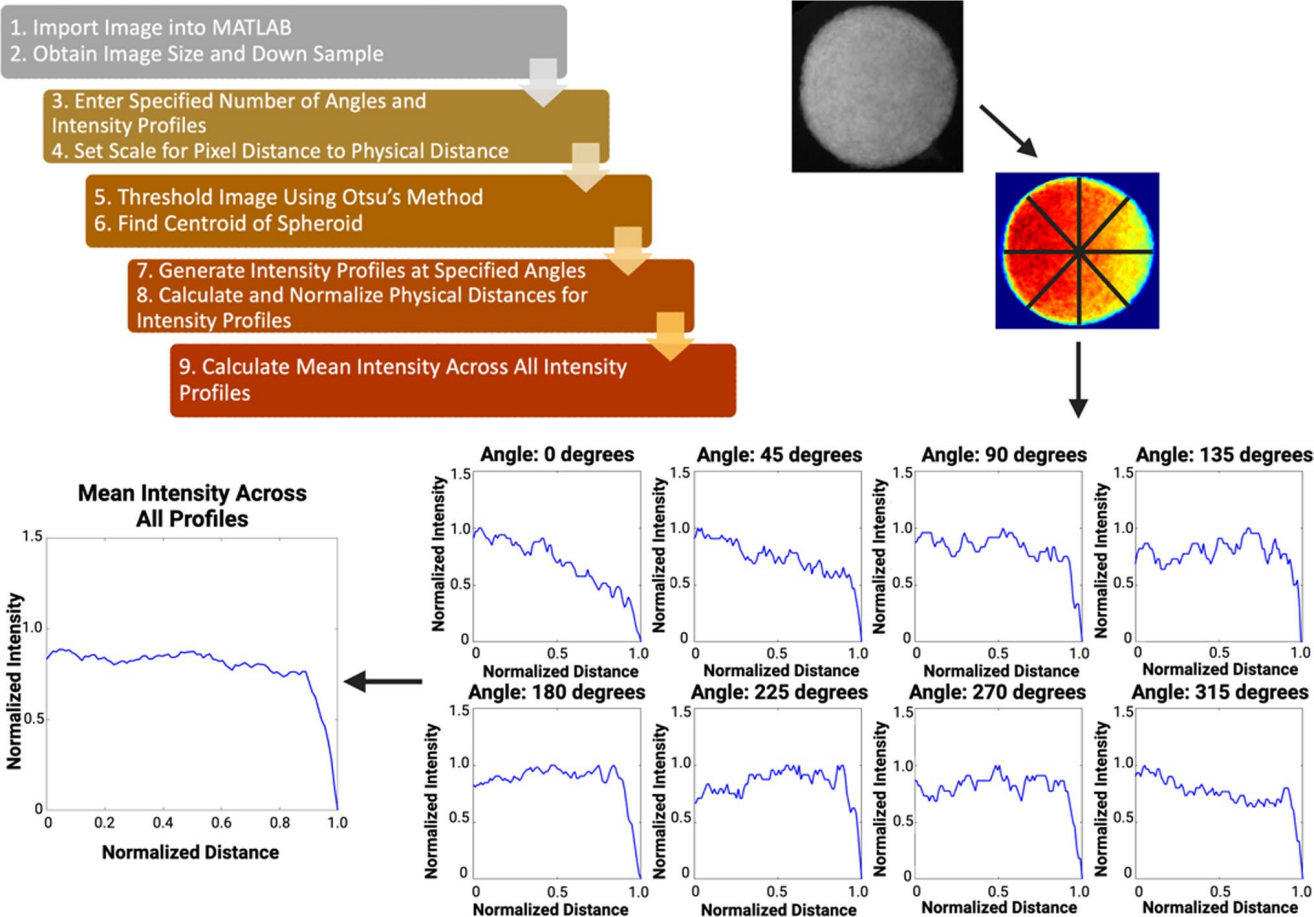
Diagram of MATLAB radial profiling code. Raw.tiff images are imported and converted to grayscale. After the number of radial profiles has been specified by the user, the centroid of the spheroid is found where intensity line profiles and a color map are generated. The individual line profiles are then used to calculate a mean line intensity profile for a spheroid. Figure was created in BioRender

A custom MATLAB script was created to analyze the intensity distributions of the immunofluorescence and metabolic images of multicellular spheroids. Briefly (Fig. 3), raw.tiff images were uploaded and converted to grayscale and then down-sampled to a 200 × 200-pixel image. The number of radial intensity profiles and points across each radial profile were specified by the user. In this study, 20 radial intensity profiles with 80 individual points along the profile were used. After the pixel-to-physical distance scale was established, a binary mask of the grayscale image was created, and the centroid of the spheroid was found. From this, the intensity profiles are generated and stored in a matrix. Subsequently, the physical distances were calculated and normalized for the intensity profiles based on the scale specified by the user. Finally, the mean intensity across all radial profiles was calculated to display a line plot for all intensity values for the spheroid. Once the mean intensities were found, the minimum and maximum values within the line profile were found and the data was normalized between 0 and1 using the following equation (Eq. 1):

1 $$Normalized\;Intensity=\frac{X_{Point\;along\;line\;profile}-X_{Minimum\;value\;across\;line\;profile}}{X_{Maximum\;value\;across\;line\;profile}-X_{Minimum\;value\;across\;line\;profile}}$$

**Fig. 4:**
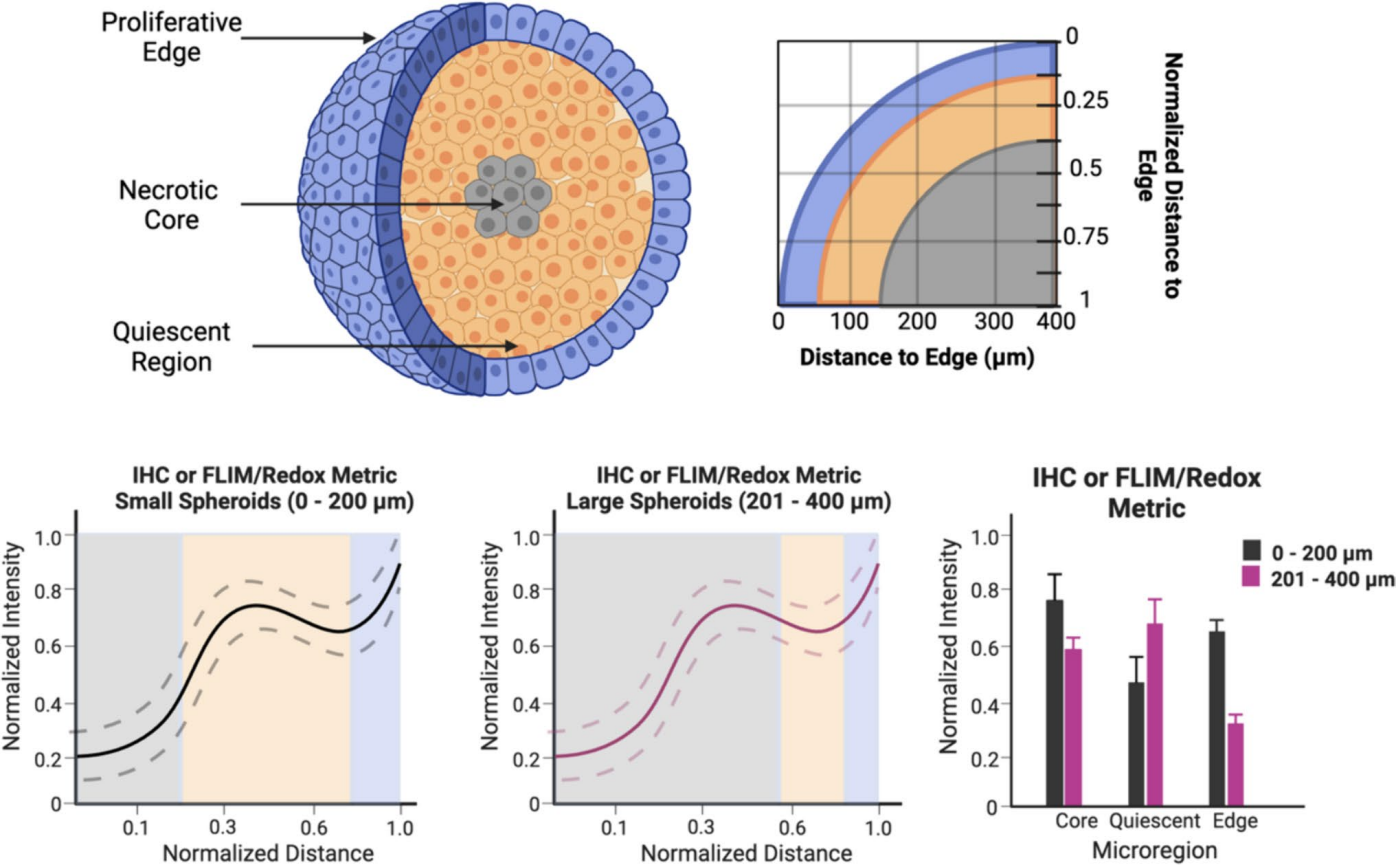
Schematic of the selection of discrete points for regional analysis. After the intensity line plots were created, three discrete points across the normalized distance was chosen to approximate the centralized location within each microregion (necrotic core, quiescent region, and proliferative edge). Figure was created in BioRender

To investigate the discrete changes in the intensity distributions at the characteristic microregions (i.e., necrotic core, quiescent, and proliferative edge), first spheroids were divided into two size ranges (0 – 200 µm and 201 – 400 µm) Fig. 4 [50]. Once the spheroids were divided into their size range categories, the size of microregions for each size range must be found. Briefly, based on the methods shown in Fig. 4, it was calculated that for spheroids ranging from 0 – 200 µm, it was found that the necrotic core and the proliferative edge each contributed to approximately 25% of the data points along the radial profile, while the quiescent region accounted for approximately 50% of the data points along the radial profile. For spheroids ranging from 201 – 400 µm, it was found that the necrotic core accounted for approximately 60% of the data points along the radial profile, while the quiescent region and the proliferative edge accounted for approximately 30% and 10%, respectively, of the radial line profile.

### Data analysis and statistics

FLIM is a microscopy technique that is used to determine the binding fraction of fluorophores, such as NADH, based on their mean lifetimes in the unbound or protein-bound states [51]. FLIM is typically performed using time-correlated single-photon counting (TCSPC), which creates a histogram of lifetime values through measuring the time between the laser pulse and the detection of an emitted photon. To get an accurate lifetime decay measurement, it is important to obtain the arrival time of thousands of photons at each pixel. To analyze NADH FLIM measurements, biexponential models are fit to the histogram at each pixel to obtain a decay curve (Eq. 2)

2 $$I\left(t\right)={I}_{0}({A}_{1}{e}^{\left({~}^{-t}\!\left/ \!{~}_{{\tau }_{1}}\right.\right)}+{A}_{2}{e}^{\left({~}^{-t}\!\left/ \!{~}_{{\tau }_{2}}\right.\right)}$$

where ${I}_{0}$ is the initial fluorescence intensity, ${\tau }_{1}$ and ${\tau }_{2}$ are the short and long lifetime components, respectively, and ${A}_{1}$(free NADH and ${A}_{2}$ (protein-bound NADH) are their respective relative contributions to the total fluorescence [52]. The ratio of ${A}_{1}$/${A}_{2}$ is frequently used as a summary statistic to describe the lifetime decay of NADH.

Pixel-wise calculations of the optical redox ratio were computed in a custom MATLAB program after normalizing fluorescent intensities based on the day-to-day variability of the lasers. A bi-exponential fit of the fluorescence lifetime decay using SPCImage provided the relative contribution of free (A1) and protein-bound (A2) NADH at each pixel. Pixel-wise mean fluorescence lifetime values, optical redox ratios, and the ratio of A1/A2 were calculated using a custom MATLAB program. An ordinary two-way analysis of variance (ANOVA) and Tukey’s multiple comparisons test were used to determine the statistical significance between microregions and spheroid diameter ranges. A *p*-value of < 0.05 is considered statistically significant.

## Results

### Combination of hanging drop and agitation culture methods produce spheroids of spherical shape with consistent diameter range

**Fig. 5:**
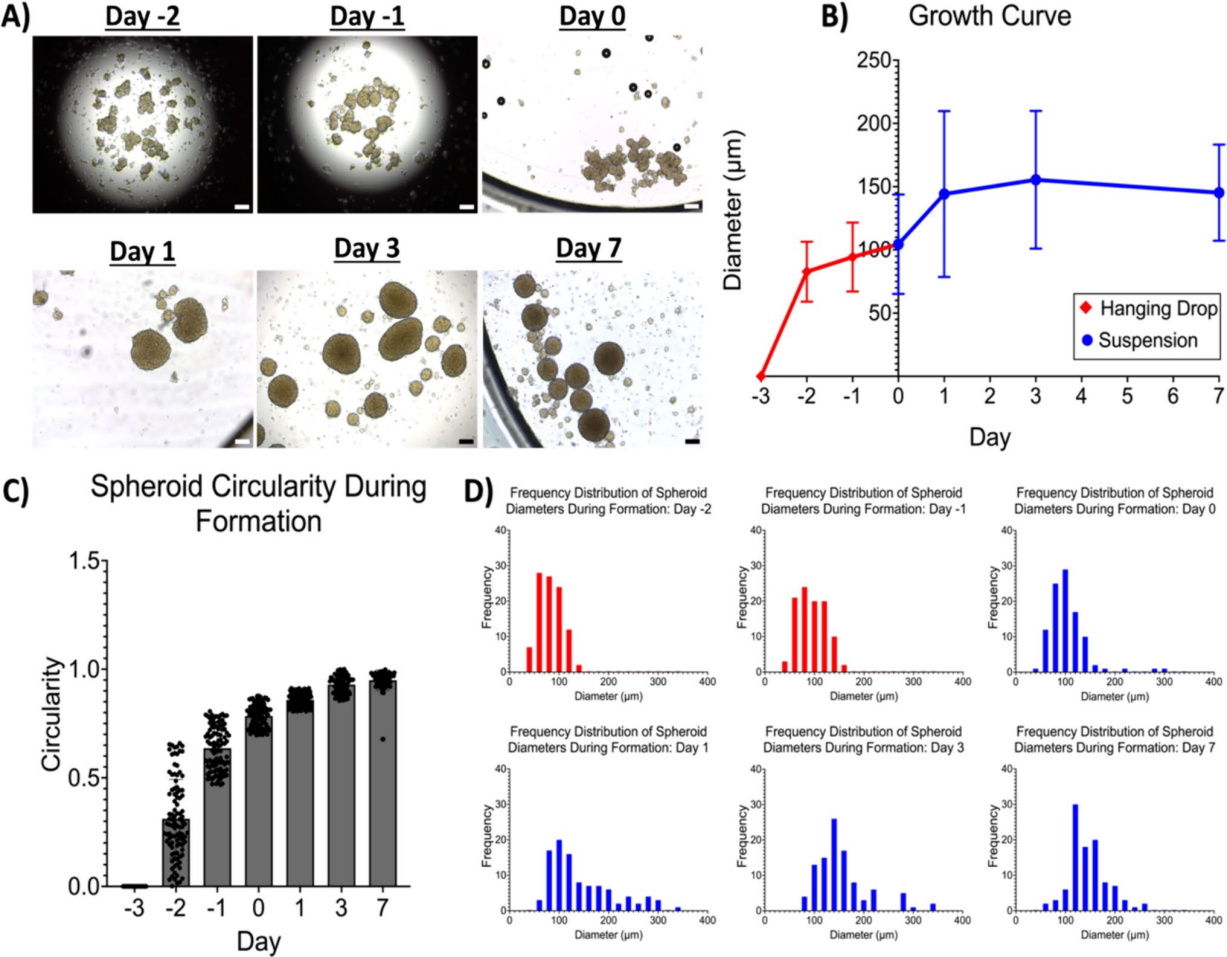
Combination of hanging drop and agitation based 3D culture methods show reproducible creation of multicellular spheroids of compact shape and consistent diameter ranges. Top: Representative wide-field microscopy images of spheroid during both growth phases. Scale bars are 20 µm. Left: Growth curve comparing changes in diameter over time. Right: Circularity measurements. *n* = 100 spheroids were analyzed. Plot was created in GraphPad Prism

To determine the reproducibility of the combined technique of hanging drop and agitation-based 3D culture of spheroids, spheroid diameters were measured and analyzed during both phases of growth. As shown in Fig. 5A-D, during the hanging drop phase, the spheroid diameter increased from 82.703 ± 23.735 µm on Day -2 to 94.33 ± 27.301 µm on Day -1, with small clusters forming. Circularity measurements confirm the increase in small clusters with an increase in circularity from 0.311 ± 0.181 on Day -2 to 0.636 ± 0.101 on Day -1. When the spheroids were transferred from the hanging drop to suspension culture, the average diameter was 104.485 ± 39.282 µm with circularity values of 0.784 ± 0.054. There was a slight increase in diameter from Day 1 (144.124 ± 65.652 µm) to Day 3 (155.463 ± 54.462 µm) with large, irregular spheroid formation. Circularity values showed an increase from 0.859 ± 0.032 on Day 1 to 0.929 ± 0.041 on Day 3. Interestingly, there was a slight decrease in diameter on Day 7 (145.207 ± 38.046 µm) with more pronounced spherical-shaped spheroid formation with circularity values of 0.950 ± 0.047. This could be attributed to the decrease in the standard deviation, indicating that the spheroids became more compact over time.

### Immunofluorescence shows significant changes in macrophage populations across micro-regions

**Fig. 6:**
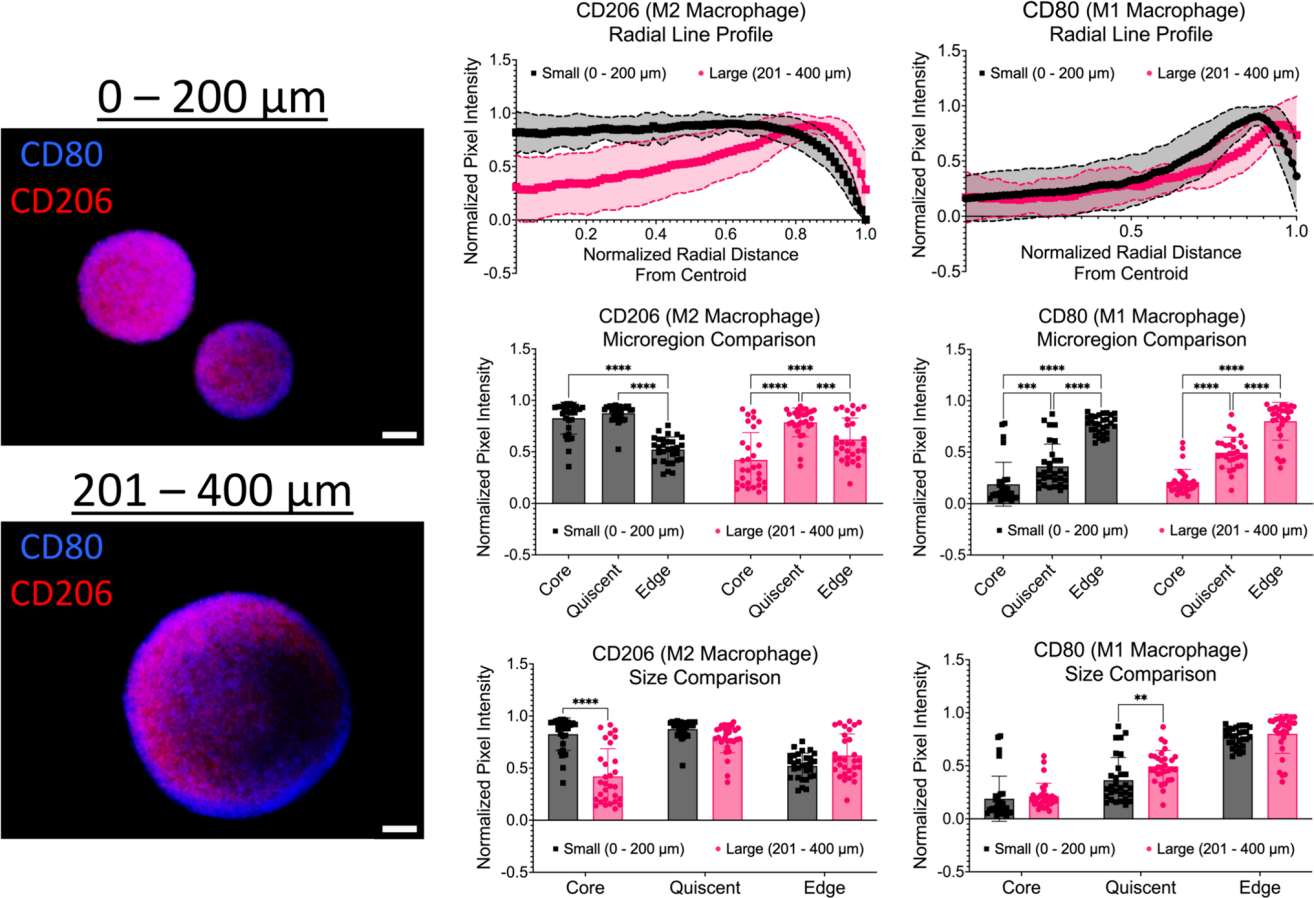
Intensity profiles show significant changes in M1 and M2 macrophage distribution across spheroid micro-regions. Left: Representative immunofluorescence images of CD80 (M1) and CD206 (M2) macrophage populations across all diameter ranges. Scale bars are 50 µm. Middle: Intensity line profiles of M1 and M2 macrophage distributions across the normalized radius of spheroids. Right: Bar plots of M1 and M2 macrophage intensities by micro-region (core vs quiescent vs proliferative edge). ** *p* ≤ 0.01; *** *p* ≤ 0.001; **** *p* ≤ 0.0001. Graphs were made in GraphPad Prism®

To determine the distribution of M1 (CD80) and M2 (CD206) macrophage populations within the spheroid model, immunofluorescence staining and imaging were performed. As shown in Fig. 6, intensity line plots of CD80 and CD206 were created to determine the distribution of M1 and M2 macrophages across the spheroid micro-regions. First, the CD80 intensities were analyzed. For small spheroids with diameters of 0 – 200 µm, the average CD80 intensities at the core, quiescent region, and proliferative edge were 0.189 ± 0.212, 0.364 ± 0.214, and 0.767 ± 0.089, respectively. Statistically significant differences were observed across all microregions (core vs quiescent (*p* = 0.0003), core vs edge and quiescent vs edge (*p* < 0.0001). For large spheroids with diameters of 201 – 400 µm, the average CD80 intensities at the core, quiescent region, and proliferative edge were 0.212 ± 0.123, 0.494 ± 0.151, and 0.801 ± 0.184, respectively. Statistically significant differences were observed across all microregions (*p* < 0.0001). In addition to investigating changes across micro-regions, changes in CD80 intensities between diameter ranges were also compared. No statistical differences were observed across the diameter ranges for CD80 intensities in the spheroid core and edge. However, a statistical difference was observed in the quiescent region between small and large spheroids (*p* = 0.0097).

Next, the CD206 intensities were explored. For small spheroids with diameters of 0 – 200 µm, the average CD206 intensities at the core, quiescent region, and proliferative edge were 0.826 ± 0.156, 0.874 ± 0.085, and 0.521 ± 0.123, respectively. Statistically significant differences were observed between the core vs edge and the quiescent vs edge (*p* < 0.0001). For large spheroids with diameters of 201 – 400 µm, the average CD80 intensities at the core, quiescent region, and proliferative edge were 0.420 ± 0.266, 0.786 ± 0.139, and 0.620 ± 0.208, respectively. Statistically significant differences were observed across all microregions (core vs quiescent and core vs edge (*p* < 0.0001) and quiescent vs edge (*p* = 0.0008). In addition to investigating changes across micro-regions, changes in CD206 intensities between diameter ranges were also compared. No statistical differences were observed across the diameter ranges for CD80 intensities in the quiescent region and the proliferative edge. However, a statistical difference was observed in the core between small and large spheroids (*p* < 0.0001). A summary data table is shown in Table 1. Taken together, the data indicates that smaller spheroids show a higher presence of M2 macrophages at the core with a larger presence of M1 macrophages at the proliferative edge, while larger spheroids show a higher presence of M2 macrophages at the quiescent region with a larger presence of M1 macrophages at the proliferative edge.

**Table 1:** Summary of normalized CD80 and CD206 intensity values

Spheroid diameter ranges	*n*	Spheroid micro-region	Normalized CD80 Intensity ± SD	Normalized CD206 Intensity ± SD
Small (0 –200 µm)	30	Core	0.189 ± 0.212	0.826 ± 0.156
		Quiescent	0.364 ± 0.214	0.874 ± 0.085
		Edge	0.767 ± 0.089	0.521 ± 0.123
Large (201 – 400 µm)	30	Core	0.212 ± 0.123	0.420 ± 0.266
		Quiescent	0.494 ± 0.151	0.786 ± 0.139
		Edge	0.801 ± 0.184	0.620 ± 0.208

### Immunofluorescence shows significant changes in cellular proliferation and apoptosis across micro-regions

**Fig. 7:**
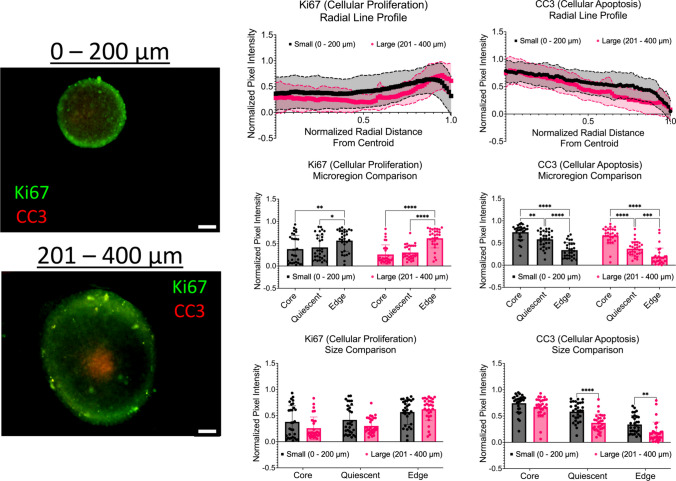
Intensity profiles show significant changes in cellular proliferation and apoptosis across spheroid micro-regions. Left: Representative immunofluorescence images of proliferation (Ki67) and apoptosis (CC3) across all diameter ranges. Scale bars are 50 µm. Middle: Intensity line profiles of proliferation and apoptosis across the normalized radius of spheroids. Right: Bar plots of Ki67 and CC3 intensities by micro-region (core vs quiescent vs proliferative edge). * *p* ≤ 0.05; ** *p* ≤ 0.01; **** *p* ≤ 0.001; **** *p* ≤ 0.0001. Graphs were made in GraphPad Prism®

To determine the distribution of cellular proliferation (Ki67) and apoptosis (CC3) within the spheroid model, immunofluorescence staining and imaging were performed. As shown in Fig. 7, intensity line plots of Ki67 and CC3 were created to determine the distribution of cellular proliferation and apoptosis across the spheroid micro-regions. First, the Ki67 intensities were analyzed. For small spheroids with a diameter range of 0 – 200 µm, the average Ki67 intensity at the core was 0.380 ± 0.308, 0.418 ± 0.276 at the quiescent region was 0.570 ± 0.240 at the proliferative edge. Statistical significance was observed between the proliferative edge and core (*p* = 0.0077), along with the quiescent region (*p* = 0.0448). For large spheroids with a diameter range of 201 – 400 µm, the average Ki67 intensity at the core was 0.259 ± 0.215, 0.303 ± 0.156 at the quiescent region was 0.622 ± 0.216 at the proliferative edge. Statistical significance was observed between the proliferative edge and core, along with the quiescent region (*p* < 0.0001). In addition to investigating changes across micro-regions, changes in Ki67 intensities between diameter ranges were also compared. No statistical differences were observed between diameter ranges.

Next, the CC3 intensities were explored. For small spheroids with a diameter range of 0 – 200 µm, the average CC3 intensities at the core, quiescent region, and proliferative edge were 0.744 ± 0.172, 0.584 ± 0.184, and 0.340 ± 0.174, respectively. Significant differences were observed across all microregions, more specifically between the core and the quiescent region (*p* = 0.0020) and the edge (*p* < 0.0001), as well as between the quiescent and the edge (*p* < 0.0001). For large spheroids with a diameter range of 201 – 400 µm, the average CC3 intensities at the core, quiescent region, and proliferative edge were 0.666 ± 0.187, 0.370 ± 0.165, and 0.192 ± 0.193, respectively. Significant differences were observed across all microregions, the quiescent and the edge (*p* = 0.0005), as well as between the core and the quiescent region and the edge (*p* < 0.0001). In addition to investigating changes across micro-regions, changes in CC3 intensities between diameter ranges were also compared. Statistical differences were observed between diameter ranges in the quiescent region (*p* < 0.0001) and the proliferative edge (*p* = 0.0051). A summary data table is shown in Table 2. Taken together, the data indicates that regardless of diameter, spheroids show a higher presence of cellular apoptosis at the core with a larger presence of cellular proliferation at the edge. Smaller diameter spheroids do show more cellular apoptosis and proliferation at the core and quiescent regions, respectively, compared to larger diameter spheroids.

**Table 2:** Summary of normalized Ki67 and CC3 intensity values

Spheroid diameter ranges	*n*	Spheroid micro-region	Normalized Ki67 Intensity ± SD	Normalized CC3 Intensity ± SD
Small (0 –200 µm)	30	Core	0.380 ± 0.308	0.744 ± 0.172
		Quiescent	0.418 ± 0.276	0.584 ± 0.184
		Edge	0.570 ± 0.240	0.340 ± 0.174
Large (201 – 400 µm)	30	Core	0.259 ± 0.215	0.666 ± 0.187
		Quiescent	0.303 ± 0.156	0.370 ± 0.165
		Edge	0.622 ± 0.216	0.192 ± 0.193

### Immunofluorescence shows significant changes in acute and chronic hypoxia across micro-regions

**Fig. 8:**
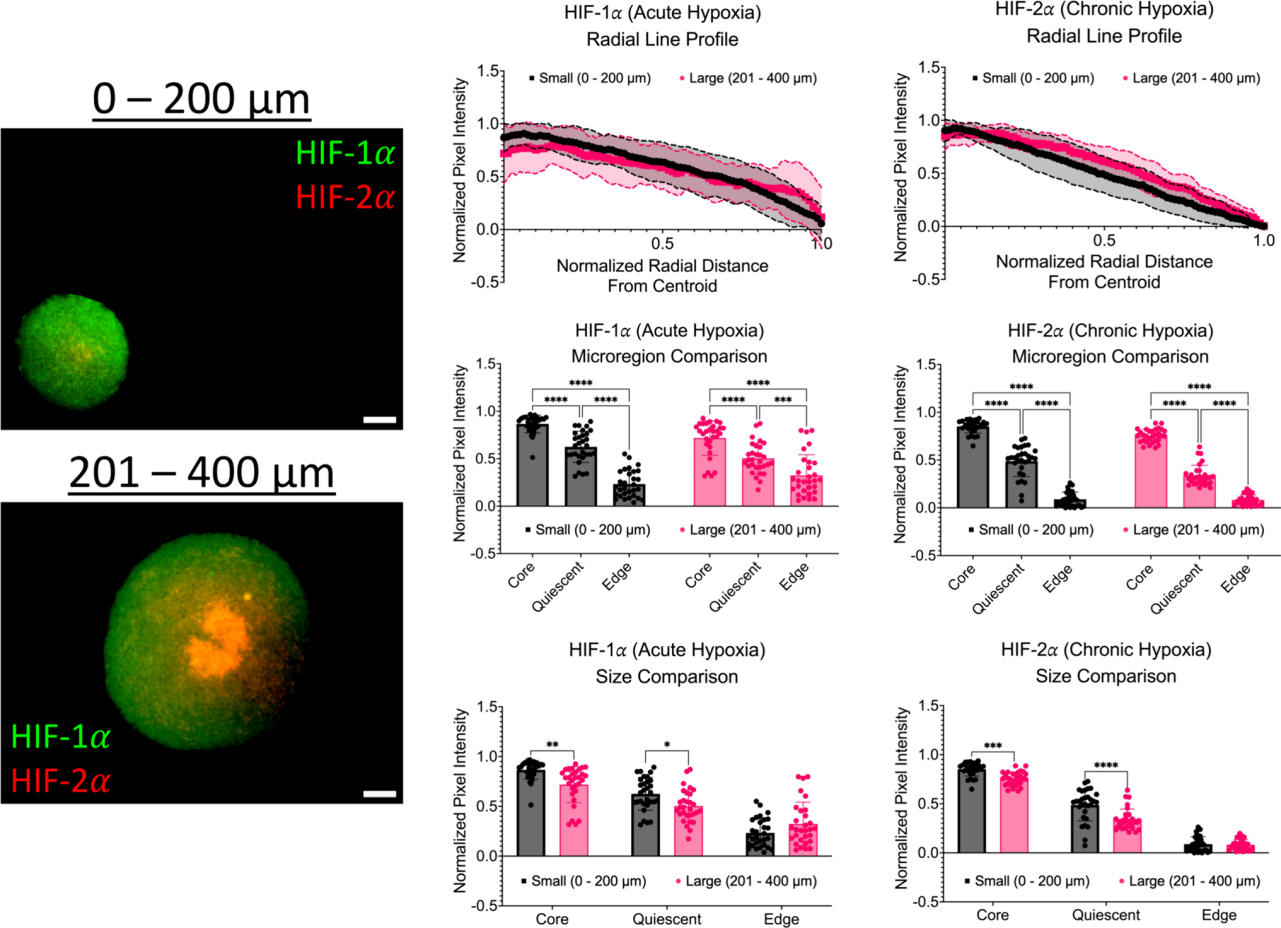
Intensity profiles show significant changes in acute and chronic hypoxia across spheroid micro-regions. Left: Representative immunofluorescence images of acute (HIF-1α) and chronic (HIF-2α) hypoxia across all diameter ranges. Scale bars are 50 µm. Middle: Intensity line profiles of acute and chronic hypoxia across the normalized radius of spheroids. Right: Bar plots of HIF-1α and HIF-2α intensities by micro-region (core vs quiescent vs proliferative edge). ** *p* ≤ 0.01: *** *p* ≤ 0.001; **** *p* ≤ 0.0001. Graphs were made in GraphPad Prism®

To determine the distribution of acute (HIF-1α) and chronic (HIF-2α) within the spheroid model, immunofluorescence staining and imaging was performed. As shown in Fig. 8, intensity line plots of HIF-1α and HIF-2α were created determine the distribution of acute and chronic hypoxia across spheroid micro-regions. First, HIF-1α intensities were analyzed. For small spheroids with a diameter range of 0 – 200 µm, the average HIF-1α intensity at the core was 0.866 ± 0.094, 0.625 ± 0.165 at the quiescent region, and 0.232 ± 0.142 at the proliferative edge. Significant differences were observed across all microregions (*p* < 0.0001). For large spheroids with a diameter range of 201 – 400 µm, the average HIF-1α intensity at the core was 0.718 ± 0.181, 0.503 ± 0.162 at the quiescent region, and 0.323 ± 0.219 at the proliferative edge. Statistical differences were observed between the core and quiescent regions and the core and the edge (*p* < 0.0001). Another statistical difference was observed between the quiescent region and the edge (*p* = 0.0001). In addition to investigating changes across micro-regions, a comparison of changes in HIF-1α intensities between diameter ranges were also performed. For HIF-1α intensities, significant differences were observed between the diameter ranges at the core (*p* = 0.0019) and the quiescent region (*p* = 0.0136).

For small spheroids with a diameter range of 0 – 200 µm, the average HIF-2α intensity at the core was 0.852 ± 0.069, 0.488 ± 0.161 at the quiescent region, and 0.089 ± 0.076 at the proliferative edge. Significant differences were observed across all microregions (*p* < 0.0001). For large spheroids with a diameter range of 201 – 400 µm, the average HIF-2α intensity at the core was 0.754 ± 0.070, 0.337 ± 0.109 at the quiescent region, and 0.081 ± 0.054 at the proliferative edge. Significant differences were observed across all microregions (*p* < 0.0001).). In addition to investigating changes across micro-regions, a comparison of changes in HIF-2α intensities between diameter ranges were also performed. For HIF-2α intensities, statistical differences were observed between the diameter ranges at the core (*p* = 0.0004) and the quiescent regions (*p* < 0.0001). A summary data table is shown in Table 3. Taken together, the data indicates that regardless of diameter, spheroids show a higher presence of both hypoxia markers (HIF-1α and HIF-2α) at the core. Smaller diameter spheroids do show higher levels of hypoxia at the core and quiescent regions, compared to larger diameter spheroids. Interestingly, larger spheroids (201 – 400 µm) do show a slightly higher presence of acute hypoxia at the proliferative edge compared to the smaller spheroids (0 – 200 µm).

**Table 3:** Summary of Normalized HIF-1α and HIF-2α Intensity Values

Spheroid diameter ranges	*n*	Spheroid micro-region	Normalized HIF-1α Intensity ± SD	Normalized HIF-2α Intensity ± SD
Small (0 –200 µm)	30	Core	0.866 ± 0.094	0.852 ± 0.069
		Quiescent	0.625 ± 0.165	0.488 ± 0.161
		Edge	0.232 ± 0.142	0.089 ± 0.076
Large (201 – 400 µm)	30	Core	0.718 ± 0.181	0.754 ± 0.070
		Quiescent	0.503 ± 0.162	0.337 ± 0.109
		Edge	0.323 ± 0.219	0.081 ± 0.054

### Intensity line profiles show discrete changes in optical redox ratio across micro-regions and across diameter ranges

**Fig. 9:**
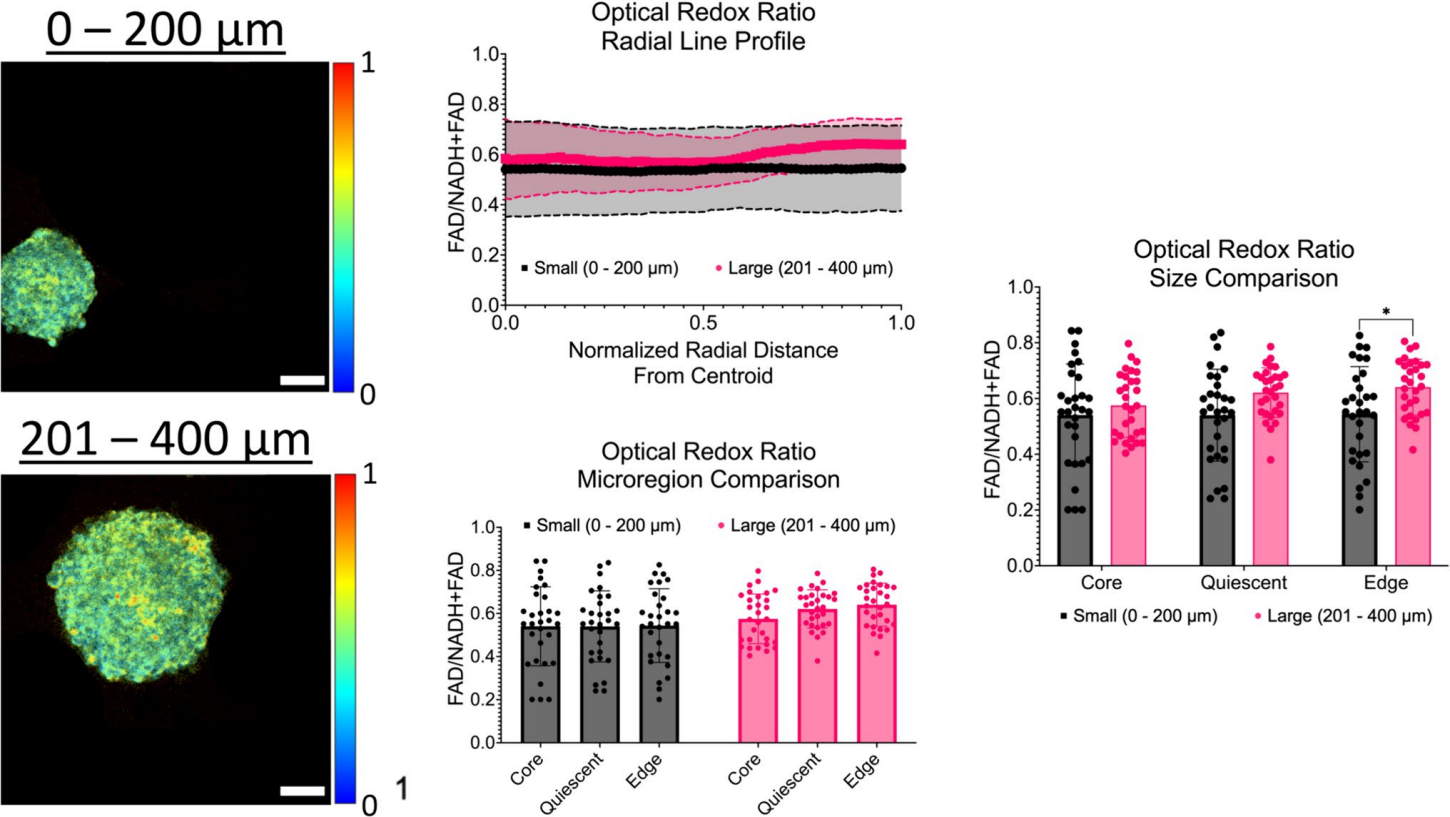
Radial profiles show discrete changes in the optical redox ratio across spheroid micro-regions. Left: Representative multiphoton images of optical redox ratio maps across all diameter ranges. Scale bars are 50 µm. Middle: Intensity line profiles of the optical redox ratio across the normalized radius of spheroids. Right: Bar plots of the optical redox ratio by micro-region (core vs quiescent vs proliferative edge). Graphs were made in GraphPad Prism®

In addition to structural characterization, changes in metabolism across spheroid micro-regions were also investigated using multiphoton imaging and fluorescence lifetime imaging (FLIM). As shown in Fig. 9, intensity line profiles of the optical redox ratio (FAD/FAD + NADH) were created. For small spheroids with a diameter range of 0 – 200 µm, the average optical redox ratios at the core, quiescent region, and proliferative edge were 0.541 ± 0.183, 0.540 ± 0.165, and 0.544 ± 0.171, respectively. No statistical differences were observed across the micro-regions. For large spheroids with a diameter range of 201 – 400 µm, the average optical redox ratios at the core was 0.575 ± 0.115, 0.621 ± 0.089 at the quiescent region, and 0.641 ± 0.100 at the proliferative edge. Again, no statistical differences were observed across the micro-regions. In addition to investigating changes across microregions, a comparison of changes in the optical redox ratio between diameter ranges was also performed. One statistical difference was observed between the diameter ranges at the proliferative edge (*p* = 0.0263). Taken together, the data suggests that larger spheroids show a higher mean optical redox value across all spheroid micro-regions compared to the small spheroids.

### Intensity line profiles show discrete changes in fluorescence lifetime metrics across micro-regions

**Fig. 10:**
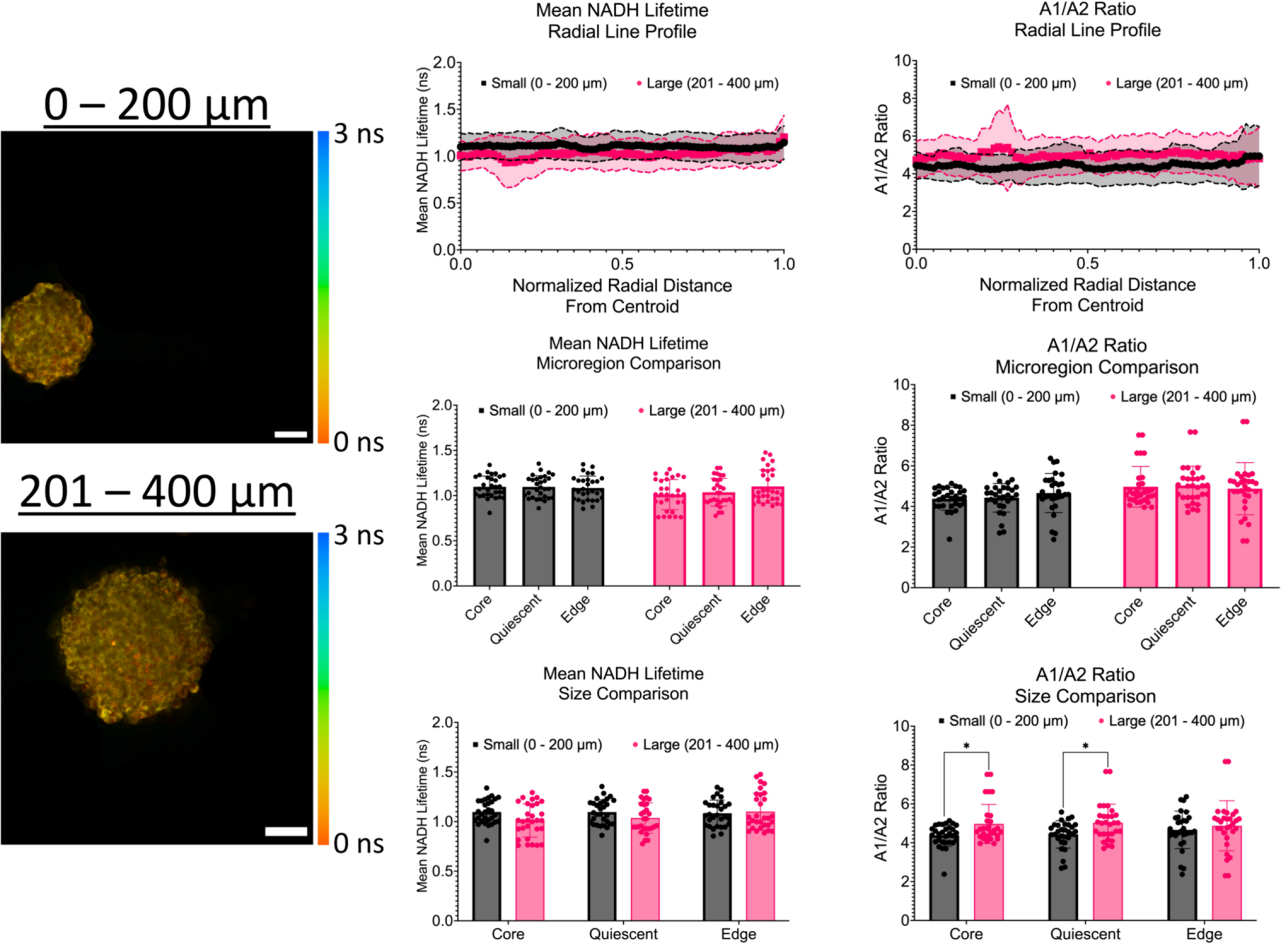
Radial profiles show discrete changes in mean NADH lifetime and A1/A2 ratio across spheroid micro-regions. Left: Representative multiphoton images of mean NADH lifetime maps across all diameter ranges. Scale bars are 50 µm. Middle: Intensity line profiles of the mean NADH lifetime and A1/A2 ratio across the normalized radius of spheroids. Right: Bar plots of the mean NADH lifetime and A1/A2 ratio by micro-region (core vs quiescent vs proliferative edge). * *p* ≤ 0.05; *** *p* ≤ 0.001. Graphs were made in GraphPad Prism®

As shown in Fig. 10, intensity line profiles of the mean NADH lifetime and A1/A2 ratios were created. First, the mean NADH lifetime was analyzed. For spheroids with a diameter range of 0 – 200 µm, the mean NADH lifetimes at the core, quiescent region, and proliferative edge were 1.097 ± 0.119 ns, 1.096 ± 0.123 ns, and 1.084 ± 0.133 ns, respectively. No statistical differences were observed across the micro-regions. For large spheroids with a diameter range of 201 – 400 µm, the mean NADH lifetime at the core was 1.011 ± 0.167 ns, 1.036 ± 0.153 ns at the quiescent region, and 1.101 ± 0.185 ns at the proliferative edge. Again, no statistical differences were observed across the micro-regions. In addition to investigating changes across micro-regions, a comparison of changes in the mean NADH lifetime between diameter ranges was also performed. No statistical differences were observed between the diameter ranges.

For the A1/A2 ratio, for small spheroids with a diameter range of 0 – 200 µm, the average A1/A2 ratio at the core was 4.379 ± 0.561, 4.429 ± 0.704 at the quiescent region, and 4.661 ± 0.963 at the proliferative edge. No statistical differences were observed across the micro-regions. For large spheroids with a diameter range of 201 – 400 µm, the average A1/A2 ratios at the core were 4.969 ± 1.002, 5.030 ± 0.954 in the quiescent region, and 4.874 ± 1.286 at the proliferative edge. Again, no statistical differences were observed across the micro-regions. In addition to investigating changes across micro-regions, a comparison of changes in the A1/A2 ratios between diameter ranges was also performed. Statistical differences between the diameter ranges were observed at the core (*p* = 0.0477) and the quiescent region (*p* = 0.0420). Altogether, smaller spheroids showed a lower mean NADH lifetime with a higher A1/A2 ratio across the micro-regions compared to larger spheroids that showed the opposite trend.

### Significant correlations were observed between diameter ranges, immunofluorescence and optical markers

**Fig. 11:**
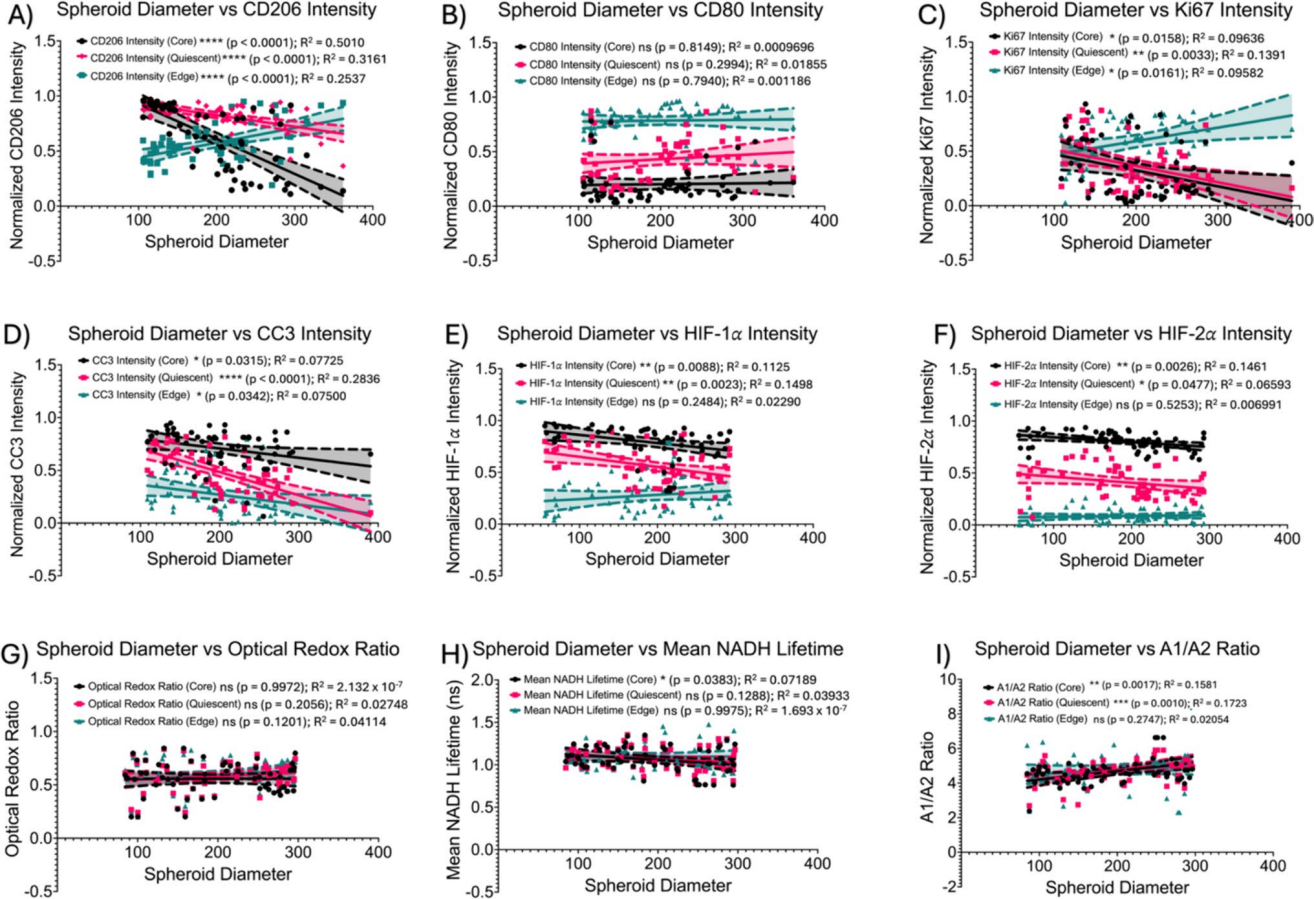
Correlations between spheroid diameter and normalized immunofluorescence intensities and metabolic imaging metrics across microregions. Spheroid Diameter vs **A** Normalized CD206 Intensity, **B** Normalized CD80 Intensity, **C** Normalized Ki67 Intensity, **D** Normalized CC3 Intensity, **E** Normalized HIF-1α Intensity, **F** Normalized HIF-2α Intensity, **G** Optical Redox Ratio, H) Mean NADH Lifetime, **I** A1/A2 Ratio. * *p* ≤ 0.05; ** *p* ≤ 0.01; *** *p* ≤ 0.001; **** *p* ≤ 0.0001. Graphs were made in GraphPad Prism®

Next, to investigate whether there is a relationship between spheroid diameter and immunofluorescence intensity and optical markers across the microregions, correlations were performed (Fig. 11). First at the core, it was observed that as spheroid diameter increases, there were significant correlations between all normalized immunofluorescence intensities, except for CD80 (Fig. 11A, C-F) and in mean NADH lifetime and the A1/A2 ratio (Fig. 11H-I). In the quiescent region, it was observed that as spheroid diameter increases, there were significant correlations between all normalized immunofluorescence intensities except for CD80 (Fig. 11A, C-F) and in the A1/A2 ratio (Fig. 11I). Lastly, at the proliferative edge, it was observed that as spheroid diameter increases, there were significant correlations between all normalized immunofluorescence intensities except for CD80, HIF-1α, and HIF-2α. No significant correlations were observed between spheroid diameter and the optical redox ratio, mean NADH lifetime, and A1/A2 ratio. A summary data tables are shown in Tables 4 and 5.

**Table 4:** Summary of correlations: immunofluorescence intensities

Region	CD206	CD80	Ki67	CC3	HIF-1α	HIF-2α
Core	R^2^ = 0.5010 *p* < 0.0001 (****)	R^2^ = 0.0009696 *p* = 0.8149 (ns)	R^2^ = 0.09636 *p* = 0.0158 (*)	R^2^ = 0.07725 *p* = 0.0315 (*)	R^2^ = 0.1125 *p* = 0.0088 (**)	R^2^ = 0.1461 *p* = 0.0026 (**)
Quiescent	R^2^ = 0.3161 *p* < 0.0001 (****)	R^2^ = 0.01855 *p* = 0.2994 (ns)	R^2^ = 0.1391 *p* = 0.0033 (**)	R^2^ = 0.3826 *p* < 0.0001 (****)	R^2^ = 0.1498 *p* = 0.0023 (**)	R^2^ = 0.06593 *p* = 0.0477 (*)
Edge	R^2^ = 0.2537 *p* < 0.0001 (****)	R^2^ = 0.001186 *p* = 0.7940 (ns)	R^2^ = 0.09582 *p* = 0.0161 (*)	R^2^ = 0.07500 *p* = 0.0342 (*)	R^2^ = 0.02290 *p* = 0.2484 (ns)	R^2^ = 0.006991 *p* = 0.5253 (ns)

**Table 5:** Summary of correlations: metabolic markers

Region	Optical redox ratio	Mean NADH lifetime	A1/A2 Ratio
Core	R^2^ = 2.132 × 10–7 *p* = 0.9972 (ns)	R^2^ = 0.07189 *p* = 0.0383 (*)	R^2^ = 0.1581 *p* = 0.0017 (**)
Quiescent	R^2^ = 0.02748 *p* = 0.2056 (ns)	R^2^ = 0.03933 *p* = 0.1288 (ns)	R^2^ = 0.1723 *p* = 0.0010 (***)
Edge	R^2^ = 0.04114 *p* = 0.1201 (ns)	R^2^ = 1.693 × 10–7 *p* = 0.9975 (ns)	R^2^ = 0.02054 *p* = 0.2747 (ns)

## Discussion

In vitro assays are crucial for studying cellular biology, offering a quick and cost-effective complement to extensive animal testing (1). However, 2D cultures fail to replicate the complex three-dimensional (3D) interactions and structures of cells in living organisms (2, 3). Three-dimensional (3D) culture systems offer a more accurate reflection of the cellular microenvironment, affecting cellular behavior and responses in ways that are more aligned with those seen in living organisms [9–13]. However, 3D cultures require robust characterization using advanced methods to ensure that they provide reliable biological data. While traditional methods, such as flow cytometry and western blotting, have been adapted for 3D cultures, challenges remain due to their reliance on cell dissociation or lysis, potentially affecting data interpretation [34, 43]. Microscopy techniques, from bright field to fluorescence microscopy, are typically used for observing spheroid growth; therefore, imaging deeper layers of spheroids can be problematic. Advanced imaging methods, such as two-photon microscopy, have been developed to better visualize the internal layers of spheroids. However, quantification of structural changes within spheroids is limited to bulk changes or discrete changes within spheroid micro-regions, providing limited data on the distribution of cell populations and, more specifically, cellular metabolism. Therefore, there is a need to develop methods that can be used to characterize spheroids across a radial profile to provide insight into the structural and metabolic characteristics of spheroids. In this study, a 3D multicellular spheroid model was created using cancer cells and macrophages, and a custom image analysis program was used to assess changes across spheroid microregions, offering improvements in understanding tumor behavior in a 3D context.

Crosstalk between cells within the tumor microenvironment (TME) plays an important role in tumor-mediated immune suppression. The increased presence of infiltrating immune cells, specifically tumor-associated macrophages (TAMs), often correlates with tumor growth and progression. However, TAMs are highly plastic in nature, which leads to high heterogeneity in solid tumors [45, 46]. The evaluation of TAMs in 2D models allows the study of TAM polarization; however, the effect of TAM infiltration or repolarization has led to a low success rate of anti-cancer macrophage-targeted immunotherapies. Therefore, quantification of macrophages within a 3D architecture that mimics the natural TME is critical. In this study, immunofluorescent surface markers for M1 (CD80) and M2 (CD206) were used to quantify the distribution of macrophages within the micro-regions of multicellular spheroids. Overall, the results indicated that M1 macrophages are typically located at the proliferative edge of a multicellular spheroid compared to M2 macrophages, which are typically located at the spheroid core as the spheroid diameter increases.

In addition to macrophage distribution, profiling of cellular proliferation (Ki67) and apoptosis (CC3) was performed. As the diameter increased, a shift in cellular proliferation occurred from the core to the proliferative edge, which has been previously observed in other studies [52, 53]. For cellular apoptosis, the prevalence of CC3 intensity was highest at the core compared to the proliferative edge, regardless of spheroid size. Hypoxia is one of the hallmarks of solid tumor development, where tumor cells can adapt to unfavorable microenvironments, causing tumors to continue to grow owing to the regulation of hypoxia-inducible factors (HIF). In this model, HIF-1α and HIF-2α were chosen based on their importance of how tumor cells can adapt to changes in oxygen gradients and spheroids are able to functionally adapt to those gradients (death and necrosis vs. adaptation and progression). As expected, our results showed that hypoxia (acute and chronic) occurs more commonly at the core than at the proliferative edge. Taken together, these immunofluorescent markers, along with the radial changes in staining intensity, show that this spheroid model can reproduce spheroids that display hallmark characteristics observed in solid tumors in vivo.

Cellular metabolism is a tightly controlled process that serves an essential function for normal cell growth and survival. However, since the identification of an altered metabolic state in diseases such as cancer, the clinical importance of understanding cancer metabolism is critical for understanding tumor biology. To better understand how 3D spheroid growth is affected by cellular metabolism, multiphoton microscopy, and fluorescence lifetime imaging (FLIM) were used to noninvasively study changes in NADH and FAD autofluorescence. In this study, three optical metrics were used to evaluate changes in NADH and FAD autofluorescence: the optical redox ratio, mean NADH lifetime, and A1/A2 ratio. Although there were no distinct trends for the optical redox ratio, as the spheroid diameter increased, there was a slight increase in the redox ratio across all micro-regions.

One of the primary challenges in intensity-based measurements of NADH and FAD, which are used for the optical redox ratio, is that these intrinsic fluorophores have a low quantum yield, making measuring the fluorescence of NADH and FAD more challenging in tissues where collagen autofluorescence may be colocalized [54]. FLIM can overcome these challenges because of its ability to extract metabolic information using a single excitation wavelength that is sensitive to changes in the molecular environment such as pH, viscosity, and temperature [54]. For the FLIM metrics, no distinct changes in mean NADH lifetime were observed for spheroids with diameters smaller than 200 µm. For spheroids with diameters larger than 201 µm, the mean NADH lifetime was shorter compared to the smaller spheroids. across the spheroid micro-regions. Looking at the A1/A2 ratio, for spheroids with diameters of 0 – 200 µm, the A1/A2 ratio was lower across spheroid micro-regions in smaller spheroids. More specifically, the increase in mean NADH lifetime taken together with the decrease in the A1/A2 ratio could indicate that larger diameter spheroids are experiencing a shift towards a more glycolytic metabolism pathway compared to smaller diameter spheroids. Previous studies have shown that multicellular spheroids have nutrient and oxygen gradients that indicate low oxygen levels with a high level of lactate compared to the edge where oxygen is present, leading to metabolic heterogeneity [54]. Even though our model did not show distinct changes in metabolism across the spheroid micro-regions, we were able to discern differences across the two chosen diameter ranges. Further studies on the heterogeneous and flexible metabolic phenotypes within tumors could help researchers and clinicians understand the influence of cellular metabolism on tumor biology and therapeutic outcomes.

Overall, 3D multicellular spheroid characterization through a novel radial profiling image analysis script allows for the evaluation of discrete structural and metabolic changes across all spheroid microregions and across diameter ranges, compared to more conventional evaluation methods that only look at discrete points. These methodologies used in this study have the potential to help evaluate traditional therapeutic regimens such as chemotherapy and radiotherapy against newer immunomodulation therapies, such as anti-CD47 and anti-PD-L1, to provide clinicians and researchers with a better understanding of anti-cancer drug efficacy.

## Data Availability

The authors declare that all data supporting the findings of this study are available within the article. A public repository of our datasets and access to the MATLAB radial profiling script can be found in this FigShare link: https://figshare.com/projects/Scaffold-Free_Development_of_Multicellular_Tumor_Spheroids_with_Spatial_Characterization_of_Structure_and_Metabolic_Radial_Profiles/208921
